# Combined and independent effects of hypoxia and tributyltin on mRNA expression and physiology of the Eastern oyster (*Crassostrea virginica*)

**DOI:** 10.1038/s41598-020-67650-x

**Published:** 2020-06-30

**Authors:** Ann Fairly Barnett, James H. Gledhill, Robert J. Griffitt, Marc Slattery, Deborah J. Gochfeld, Kristine L. Willett

**Affiliations:** 1 Division of Environmental Toxicology, Department of BioMolecular Sciences, University of Mississippi, P.O. Box 1848, University, MS 38677 USA; 2 School of Ocean Science and Engineering, University of Southern Mississippi, 703 East Beach Road, Ocean Springs, MS 39564 USA; 3 National Center for Natural Products Research, University of Mississippi, P.O. Box 1848, University, MS 38677 USA

**Keywords:** Molecular biology, Environmental sciences, Ocean sciences

## Abstract

Oyster reefs are vital to estuarine health, but they experience multiple stressors and globally declining populations. This study examined effects of hypoxia and tributyltin (TBT) on adult Eastern oysters (*Crassostrea virginica*) exposed either in the laboratory or the field following a natural hypoxic event. In the laboratory, oysters were exposed to either hypoxia followed by a recovery period, or to hypoxia combined with TBT. mRNA expression of *HIF1-α* and *Tβ-4* along with hemocyte counts, biomarkers of hypoxic stress and immune health, respectively, were measured. In field-deployed oysters, *HIF1-α* and *Tβ-4* expression increased, while no effect on hemocytes was observed. In contrast, after 6 and 8 days of laboratory-based hypoxia exposure, both *Tβ-4* expression and hemocyte counts declined. After 8 days of exposure to hypoxia + TBT, oysters substantially up-regulated *HIF1-α* and down-regulated *Tβ-4*, although hemocyte counts were unaffected. Results suggest that hypoxic exposure induces immunosuppression which could increase vulnerability to pathogens.

## Introduction

Oysters are important ecosystem engineers, providing essential fish habitats that are vital to the health of many estuarine ecosystems worldwide^1, 2^. Oyster reefs provide numerous critical ecosystem services, including enhancing biodiversity, improving water quality, serving as nursery habitat for commercially important fisheries, stabilizing shorelines, and providing a significant economic resource for coastal communities^2–6^. Despite their ecological and economic importance, it is estimated that oyster populations have suffered losses of approximately 85% worldwide in recent years, classifying oyster reefs as one of the most heavily impacted ecosystems in the world^3^.

Extensive restoration efforts are currently underway to restore oyster reefs in the Gulf of Mexico and other areas of the United States, where these ecosystems once flourished. However, the success of these restoration efforts has lagged far behind that of other estuarine ecosystems^7^. Historically, overharvesting played a significant role in the decline of oyster populations^8^, but oyster reef recovery and resilience are also limited by poor water quality, disease, and predation^9,10^. In addition, environmental contaminants (e.g., metals, pharmaceuticals, agricultural runoff) have the potential to bioaccumulate in these filter-feeding organisms^11,12^. Due to their proximity to land and sources of freshwater, estuaries often experience large fluctuations in environmental conditions, such as temperature, salinity, and dissolved oxygen (DO), that can have negative short-term impacts on oyster reefs^13^. The complexity of estuarine systems may result in oysters often facing combinations of stressors simultaneously, and these can have greater detrimental effects than each stressor independently^13,14^. Therefore, understanding the impacts of multiple stressors, both alone and in combination, is essential to determine their impacts on aquatic organisms.

Hypoxia, an indirect effect of eutrophication, is increasingly affecting estuarine environments^15^. Hypoxia occurs when the DO concentration drops below 2 mg/L^16,17^. Hypoxia negatively impacts the microbiomes of adult oysters, affecting their energy dynamics and increasing vulnerability to pathogens^18^. Long-term hypoxic events can kill large numbers of marine organisms, including oysters, but even short-term hypoxic events can cause detrimental sub-lethal impacts to oysters such as reduced fecundity, growth rates, and immunosuppression^19–22^.

Tributyltin (TBT), once universally used as an antifouling paint on ships, is now known to have widespread toxic effects on non-target marine organisms, such as incidence of imposex in the marine gastropod *Nucella lapillus*, in addition to the targeted biofouling communities^23^. The widespread use of TBT was based on its biocidal activity, particularly against molluscs, and it is now known to be one of the most toxic pollutants to early life stages of bivalves^24,25^. While not generally lethal to adult oysters, TBT can increase their susceptibility to calcification anomalies, levels of the protozoan pathogen *Perkinsus marinus*, which causes Dermo, a disease commonly associated with mass mortalities in oyster populations, and incidence of imposex, which can result in reproductive failure^26,27^. Effects of TBT can be compounded in the presence of other environmental stressors. For example, at sublethal concentrations of TBT, oysters experienced increased mortality and TBT bioaccumulation when also exposed to hypoxic conditions^28^. Although its use as a biocide was banned by the International Maritime Organization in 2003, and it has more recently undergone further global bans, enforcement is not universal^23^. TBT can persist in marine sediments for several years, with the potential for resuspension under certain environmental conditions^27,29^. Recent data on butyltin concentrations ranged from 16.6–25.8 ng/g dry weight of TBT in oyster tissues collected from the Mississippi Sound^30^.

In this study, the effects of hypoxia in the presence and absence of TBT on the Eastern oyster (*Crassostrea virginica*) were studied in three contexts, specifically: a field deployment in the Mississippi Sound, during which oysters experienced a natural hypoxic event; a lab-based experiment wherein oysters were exposed to three durations of hypoxia, followed by a normoxic recovery period; and a lab-based exposure to TBT and hypoxia in combination. In addition to mortality, several sublethal responses to these stressors were assessed. Effects were measured by analyzing messenger ribonucleic acid (mRNA) expression of hypoxia inducible factor-1α (*HIF1-α*), which is known to be up-regulated under hypoxic stress^31^. Immune function was assessed by analyzing mRNA expression of thymosin-beta-4 (*TB-4*), which is involved in hemocyte production and mobilization^32^. Additionally, total circulating hemocytes were counted to further assess immune function, because hemocytes are phagocytic cells that provide defense against pathogens^28^. We hypothesized that oysters subjected to longer durations of hypoxia would have enhanced *HIF1-α* expression and suppressed immune function compared to controls, and that exposure of oysters to combined stressors would elicit greater responses than either stressor alone.

## Materials and methods

### Oyster collection and maintenance

Oysters (*C. virginica*) used in both field and laboratory experiments were obtained from the Auburn University Shellfish Laboratory’s farm in Bayou La Batre (Dauphin Island, AL) in early July 2018. The oysters were transported to the School of Ocean Science and Engineering Gulf Coast Research Laboratory (GCRL; Ocean Springs, MS) and placed in flow-through holding tanks supplied with water from Davis Bayou, where salinity and temperature was similar to their site of collection, ranging from 15–18 ppt and 17–20 °C, respectively. Oysters were fed ad libitum each day with Shellfish Diet 1,800 (Reed Mariculture, Campbell, CA, USA). Oysters were kept in holding tanks for one week before being transferred to exposure aquaria at GCRL to begin the hypoxia alone exposure (Experiment 1), and three weeks before being transferred to the exposure aquaria for the hypoxia + TBT exposure (Experiment 2). They were maintained for two weeks before being transferred to oyster sensor platforms for the field deployment.

### Field deployment

For the field deployment, 40 oysters were randomly selected from the holding tanks, and 20 were placed in each of two oyster sensor platforms^33^, and submerged at 1 m depth off of the dock near GCRL (Marsh Point, MS: 30° 23′ 31.488″ N, 88˚48′27.226″W) for three weeks, beginning on 20 July 2018. The sensor platforms were equipped with water quality sensors (HOBOware loggers; Onset Computer, Bourne, MA, USA) that measured dissolved oxygen, conductivity, and temperature for the duration of deployment, and housed oysters on trays enclosed within crates on each platform, with holes to allow adequate water flow. At the beginning of the experiment, oysters were taken directly from GCRL holding tanks and sampled as time-zero (T-0) oysters (n = 5). Oysters were sampled from the platforms on day 14 (T-1) and day 21 (T-2) of deployment (n = 5 from each platform on each day). Oysters were sacrificed to provide gill tissue for analysis of mRNA expression (2.5) and hemolymph for total circulating hemocyte counts (2.6). At T-2, a subset (n = 3) of the remaining oysters was used for TBT tissue accumulation analysis (2.4).

### Experimental design

#### Laboratory exposure system

The hypoxia and TBT exposures were conducted at the University of Southern Mississippi’s Toxicology Laboratory. Each exposure was performed in 3–4 replicate glass aquaria as a fully flow through exposure, with automated control over temperature, salinity, dissolved oxygen, and contaminant concentration. Temperature was controlled by circulating water around the exposure tanks through a heat exchanger. Salinity was controlled by automatically mixing artificial seawater (30 ppt) and fresh, unchlorinated well water to achieve the desired salinity. Hypoxic conditions were generated by bubbling N_2_ gas into treatment water in header tanks, which was then pumped into exposure tanks at a controlled rate. For the TBT exposure, TBT was provided from a stock solution of diluted TBT chloride (TBTCl 96%, Sigma-Aldrich #T50202) added to a header tank that was pumped into exposure tanks to produce a final concentration of approximately 80 ng/L. TBT exposure concentration was selected based on a previous study of adult oysters^28^. Replacement water for each treatment flowed into each replicate aquarium at 2 L/h. Effluent water was passed through a 7-point filtration system before release.

#### Experiment 1: hypoxia-recovery

**Figure 1:**
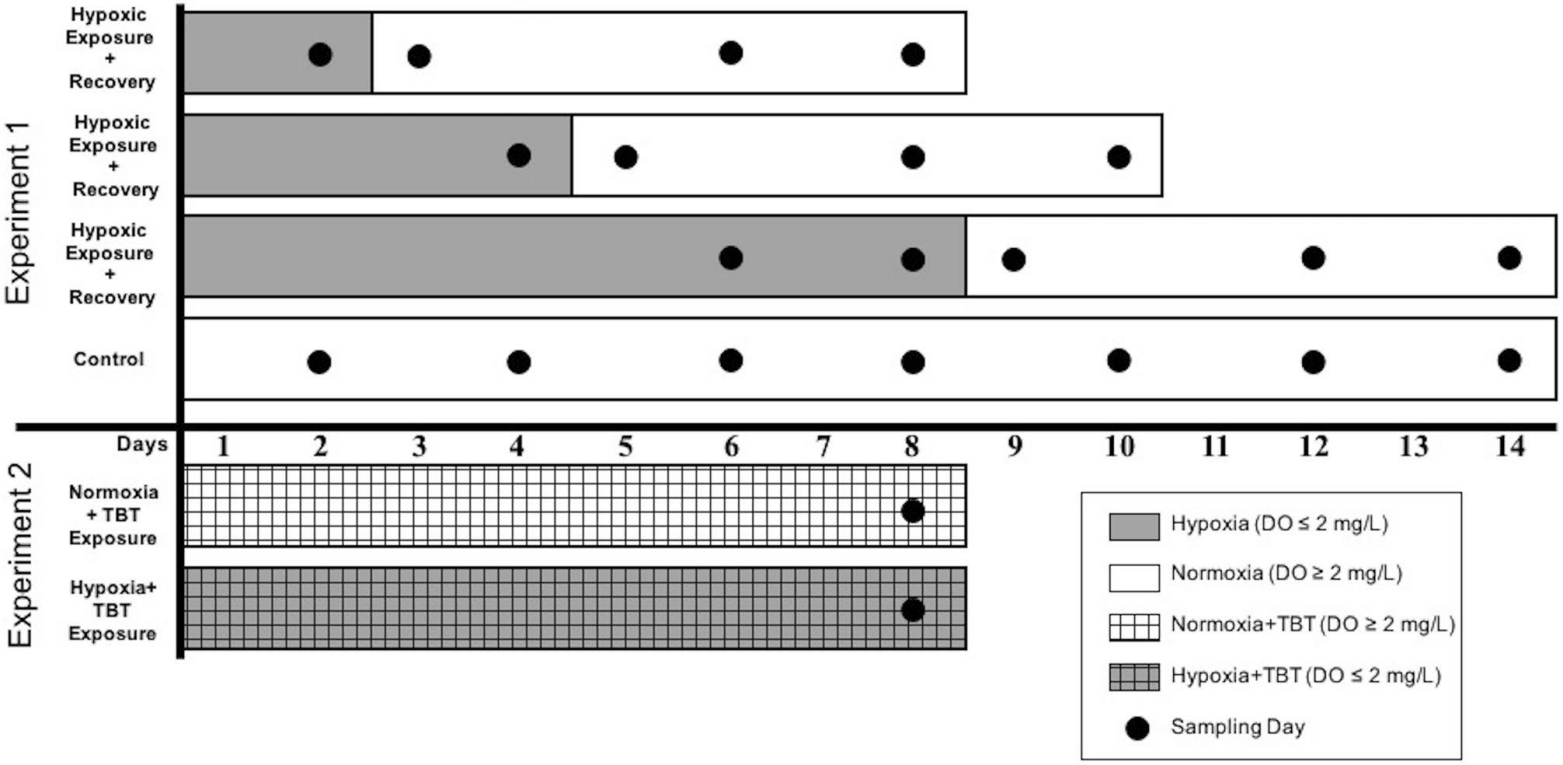
Experimental design of Experiments 1 and 2. In Experiment 1 (hypoxia-recovery), oysters were exposed to three durations of hypoxia, followed by 6 days of recovery at normoxic conditions. Control tanks remained normoxic for the duration of the experiment. Each tank (n = 3 per treatment) started with 20 oysters, with two oysters sampled on each sampling day (represented by black dots). Experiment 2 consisted of two treatments: normoxia + TBT or hypoxia + TBT. Each tank (n = 8 per treatment) started with 20 oysters and three were sampled on day 8 from every tank.

Oysters were randomly selected from holding tanks, transferred into exposure tanks, and allowed to acclimate for two days. Treatment groups were subjected to one of three durations of hypoxia (DO < 2 mg/L): 2, 4, or 8 days (Fig. 1), beginning on 14 July 2018. Each treatment was then followed by a 6-day recovery period in normoxic conditions (DO ≥ 8 mg/L). The control group was maintained in normoxic conditions for 14 days. Each of the four treatments consisted of three replicate 65 L tanks maintained at a temperature of 18 °C and salinity of 151 ppt. Each tank started with 20 oysters, and two oysters were removed from all tanks on each sampling day so that oyster density remained consistent across tanks on each day of the experiment. Upon removal from exposure tanks, gill and hemolymph samples were collected from sacrificed oysters for analysis of mRNA expression (2.5) and counts of total circulating hemocytes (2.6), respectively.

#### *Experiment 2: hypoxia* + *TBT*

Oysters were randomly selected from holding tanks and transferred to replicate 65 L exposure tanks to acclimate for two days. Beginning on 1 August 2018, oysters were exposed to independent and combined hypoxia and TBT treatments in a factorial design for 8 days, with the following conditions: TBT (80 ng/L), hypoxia (DO < 2 mg/L), and normoxic conditions (DO ≥ 8 mg/L). Hypoxic conditions and TBT dosing were generated as described in 2.3.1. Initially, each treatment consisted of four replicate tanks containing 20 oysters, which were maintained at the same temperature and salinity conditions as in Experiment 1 (2.3.2). At day 0 and day 8, samples (n = 3) were collected from each exposure tank to quantify TBT tissue accumulation. Upon later analysis, it was found that oysters in all treatments of Experiment 2 had inadvertently accumulated similar concentrations of TBT (mean ± standard error; 37.7 ± 4.11, 34.8 ± 6.34, 23.8 ± 5.18, 44.5 ± 12.7 ng Sn g^−1^ dry wt for normoxic control, hypoxia, noromoxia + TBT, and hypoxia + TBT, respectively). Therefore, data were analyzed by combining the planned control (normoxia/no TBT) and normoxia + TBT oysters into a “normoxia + TBT” treatment, and the planned hypoxia and hypoxia + TBT oysters into a “hypoxia + TBT” treatment, each consisting of 8 replicate tanks. While this does not allow for an assessment of the effects of TBT exposure independently, it does allow for comparison of the effect of an environmentally relevant dose of TBT on hypoxia-induced effects in oysters. On Day 8, gill tissue was collected for mRNA expression analysis (2.5; n = 8 per treatment) and hemolymph was extracted for hemocyte counts (2.6; n = 6 per treatment).

### Tributyltin tissue accumulation analysis

Whole body tissue samples collected from oysters at T-0 (n = 3), T-2 in the field deployment (n = 3), and on day 8 in Experiment 2 (n = 12) were sent to B&B Laboratories (TDI-Brooks International, Inc., College Station, TX) for determination of butyltins (BTs; SOPs B&B 1,012, 1,033, and 1,068 for sample preparation, extraction, and BTs, respectively). Each sample was individually extracted and analyzed for BTs by capillary gas chromatography/mass spectrometry (GC/MS) in selected ion monitoring mode (SIM). Briefly, to prepare samples, an IKA Werke tissuemizer was used to extract 0.83–1.66 g wet tissue three times in 60 mL of 0.1% tropolone in dichloromethane. BT compounds were converted to their hexylated homologues, cleaned up with Florisil/5 g Silica gel column chromatography and concentrated for analysis. Calibration solutions were prepared by diluting reference standard solutions containing the analytes of interest. For mono-, di-, tri-, and tetra-butyltin, a linear curve was generated based on the internal standard and the analyte response area and concentration ratios. Concentrations were reported in ng Sn g^−1^ dry oyster tissue.

### *mRNA expression–reverse transcription–quantitative polymerase chain reaction *(*RT-qPCR*)

Following collection of approximately 1 mm^2^ of gill tissue removed using stainless steel forceps and dissecting scissors, the tissue was placed in RNAlater™ (Invitrogen #AM7021) and stored at -80 °C until processing. RNA isolation was achieved using TRIzol™ reagent (Invitrogen #A33251) and RNase-Free DNase kit (Qiagen #74,004) according to the manufacturer’s protocol. RNA extract was quantified and assessed for purity using a NanoDrop 2000 spectrophotometer. Following quantification, RNA (250 ng) was reverse transcribed to cDNA sub-stocks at a final concentration of 10 ng/µL following manufacturer’s protocol (Invitrogen #4,304,134). RT-qPCR was performed using an Applied Biosystems 7,200 using SYBR™ Green PCR Master Mix (Applied Biosystems #4,309,155) with the following parameters: 95 °C for 10 min, then 40 cycles of 95 °C for 15 s and 60 °C for 1 min, followed by 95 °C for 15 s, 60 °C for 1 min, and 95 °C for 15 s to generate a dissociation curve. Primer optimization was completed using the same instrumentation and procedure. Primer sequences and efficiencies are listed in Table 1. All samples were screened in duplicate and fold-change was calculated using the 2^−ΔΔCT^ method^34^. *18 s* (Experiment 1) and elongation factor 1 alpha (*EF1-α*) (field deployment and Experiment 2) were utilized as reference genes for mRNA expression analysis.

**Table 1:** RT-qPCR primers used in this study.

Gene	Accession	Primer sequence	r^2^	Efficiency %
*Tβ-4*	LOC111123286	F: 5′- TCT GTG ATT GTG GGC TGT GTT -3′	0.995	101.01
		R: 5′- TGG TGG GTA GAG GGT TCT TCT -3′		
*HIF1-α*	XM_022475425	F: 5′-ACC AGT GAC GCC CTG TTC TC-3′	0.999	94.67
		R: 5′-ACA GAC TCG GTG CGA CCA A-3′		
*EF1-α*	XM_022472315	F: 5′-GGT ATC TCG GCA AAC GGA CA-3′	0.99	100.76
		R: 5′-TTC GTT GAA ACG GCT CTC AC-3′		
*18 s*	EU 660,792	F: 5′-CCG TCC GTT TTG GTG ACT CT-3′	0.991	96.06
		**R:** 5′-CCT TGG ATG TGG TAG CCG TT-3′		

### Total hemocyte counts

Hemolymph was extracted from the pericardial cavity of each oyster using a sterile 16-gauge syringe and transferred to a 1.7 mL centrifuge tube. Hemolymph was diluted 1:1 with 10% formalin for preservation. Hemocyte counts were performed using either a Benchtop B3 series FlowCAM particle imaging system (Fluid Imaging Technologies, Inc., Yarmouth, ME, USA). (for the field deployment and Experiment 1) or a hemocytometer (Experiment 2). The FlowCAM was equipped with a 300 µm flow cell and a 20 × objective lens, and was set to auto-image mode, in which photographs were taken of cells at 20 frames per second at a constant flow rate of 0.01 mL/min. For the field deployment and Experiment 1, two technical replicates were counted for each sample (100 μL each) and averaged to obtain a final count for each individual oyster. Hemocytometer (Hausser Scientific) counts (five per sample) were performed by filling both counting chambers with hemolymph (0.1 µL per side) and counting the hemocytes in each of the four 1 mm^2^ corners of the chambers.

### Prevalence of P. marinus cells

A subset of oysters (n = 16) from holding tanks and one oyster from each tank at the end of Experiment 2 (n = 16) were analyzed for prevalence of *P. marinus* cells, which cause Dermo disease, following the standard Ray’s Fluid Thioglycollate Medium (RFTM) protocol^35,36^.

### Data analysis

Prior to data analysis, data were tested for normality and equal variance using Shapiro–Wilk’s and Brown-Forsythe’s tests, respectively, in SigmaPlot version 14.0. Data from the field deployment (mRNA expression and hemocyte counts) were analyzed using one-way analyses of variance (ANOVA) (n = 5 per time point) followed by Tukey’s post hoc tests, if appropriate. For laboratory experiments, all oysters removed from the same tank on each day were pooled for each analysis and tanks were used as replicates. For Experiment 1 (Hypoxia-recovery), mRNA expression and hemocyte counts were analyzed using two-way ANOVAs with time and treatment as factors, followed by Tukey’s post hoc tests, if appropriate. Control oysters were not sampled on the first day of recovery, but to evaluate acute changes in mRNA expression, treated oysters at 24 h of recovery (days 3, 5 and 9) were compared to those from the final hypoxic time point (days 2, 4 and 8, respectively) using unpaired *t*-tests. Hemocyte counts were also analyzed using unpaired *t*-tests to compare exposed oysters to controls (n = 2–3 tanks per treatment) on each day. For Experiment 2 (Hypoxia + TBT), mRNA expression (n = 8) on day 8 was analyzed using unpaired *t*-tests between treatments and hemocyte counts (n = 6) on day 8 were analyzed by one-way ANOVA followed by Tukey’s post-hoc test. For each experiment, mRNA expression was standardized to that of the T-0 oysters, and log_2_ values were used in the statistical analysis. mRNA expression results are reported in graphs as log_2_ (2^−ΔΔCT^). Statistical significance was determined at p ≤ 0.05.

## Results

### Field deployment

#### In situ* water quality data*

**Figure 2:**
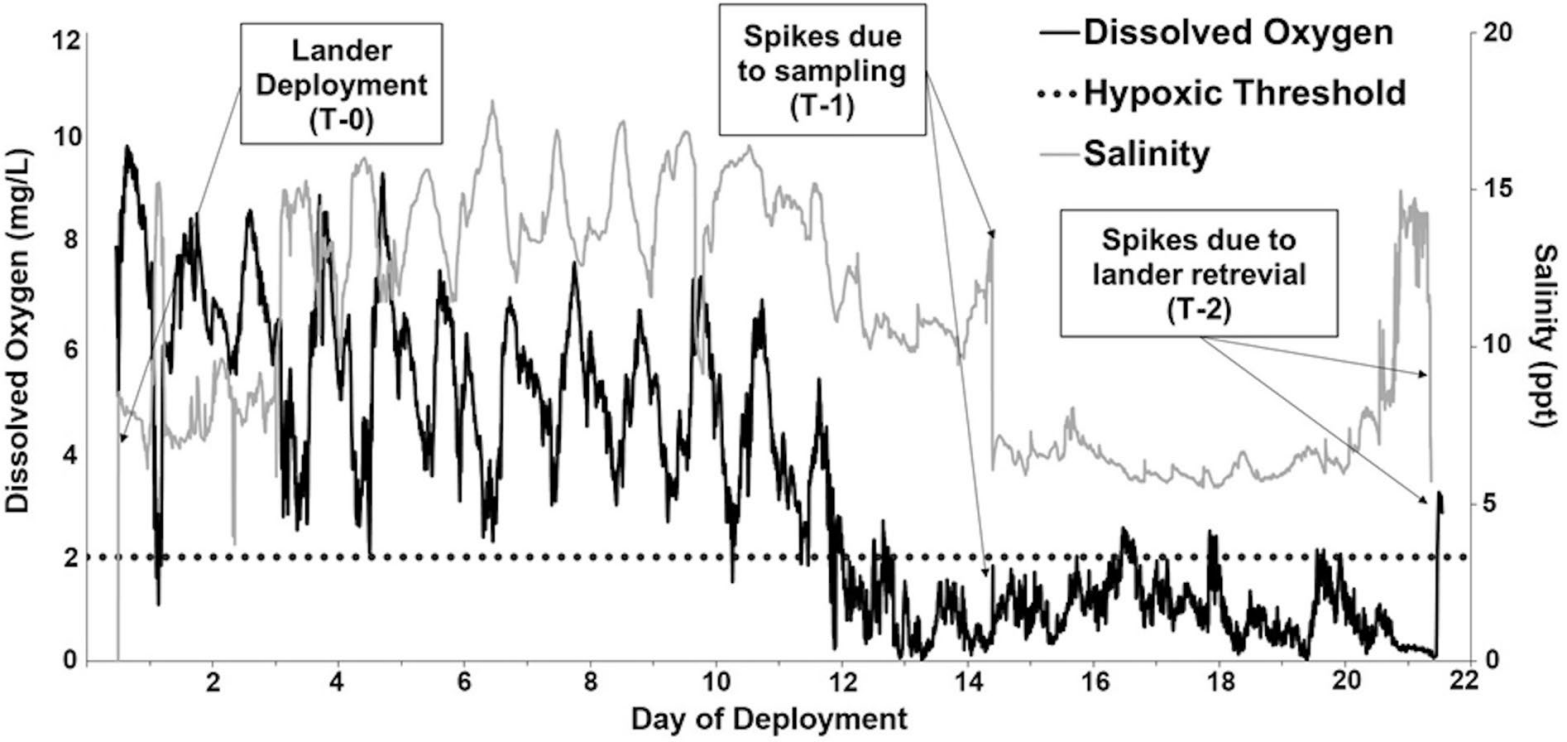
Dissolved oxygen (mg/L) and salinity (ppt) measured by in situ sensors on oyster sensor platform near Marsh Point, MS, during three-week field deployment (20 July–10 August 2018). Note the natural hypoxic event that occurred when DO fell below the hypoxic threshold (< 2 mg/L) on day 12. Oysters were sampled prior to deployment (T-0), on day 14 (T-1) and on day 21 (T-2).

An oyster sensor platform was deployed for three weeks near Marsh Point, MS from 20 July to 10 August 2018, which overlapped with the laboratory experiments. During deployment, the in situ water quality sensor data (Fig. 2) indicated fluctuating diel DO concentrations for the first 11 days of deployment. On day 12, DO dropped below 2 mg/L and remained near or below the hypoxic threshold for the remainder of the deployment. There was a concurrent decrease in salinity to a minimum of 4 ppt during the hypoxic event, with a subsequent return to 12–15 ppt near the end of the deployment, even while DO remained low.

#### Tissue accumulation of tin compounds

Tissue from oysters collected at T-0 and T-2 was sent to B&B Laboratories for determination of mono-, di-, tri-, and tetra-butyltins and are reported in Table 2. Two of the three tissue samples measured below detection limit (< 9.95 ng Sn g^−1^ dry wt oyster tissue) for mono-, di-, and tri-butyltins, while the third sample measured 22.5 ng Sn g^−1^ of monobutyltin, 41.8 ng Sn g^−1^ of dibutyltin, and below detection limit for tributyltin. All three samples measured below detection limit for tetrabutyltin.

**Table 2:** Mean concentrations of mono-, di-, and tri-butyltin in samples collected at T-0 (n = 3) and T-2 of field deployment (n = 3), and in each treatment of Experiment 2 on day 8 (n = 12).

	Mean ± SE concentration of BTs in oyster tissue (ng Sn g^−1^ dry tissue wt) and percent (%) of total Sn						Total (ng Sn g^−1^ dry tissue wt)
Treatment (n = 3)	Monobutyltin		Dibutyltin		Tributyltin		
Control	541.1 ± 332.5	22%	1835.5 ± 829.6	76%	37.7 ± 4.11	2%	2,414
Normoxia + TBT	163.3 ± 62.5	18%	701.3 ± 246.5	79%	23.8 ± 5.18	3%	888
Hypoxia	217.5 ± 17.8	25%	631.03 ± 13.5	71%	34.8 ± 6.34	4%	883
Hypoxia + TBT	546.1 ± 236.2	20%	2,148.9 ± 766.1	78%	44.5 ± 12.71	2%	2,740
Time zero	60.4 ± 6.5	16%	305.3 ± 33	82%	5.25 ± 0.48	1%	371.2
Field deployment	7.5 ± 7.5	54%	16.7 ± 12.6	40%	2.84 ± 0.43	7%	42.03

All samples measured below the detection limit (< 9.95 ng Sn g^−1^ dry wt) for tetrabutyltins.

#### mRNA expression

**Figure 3:**
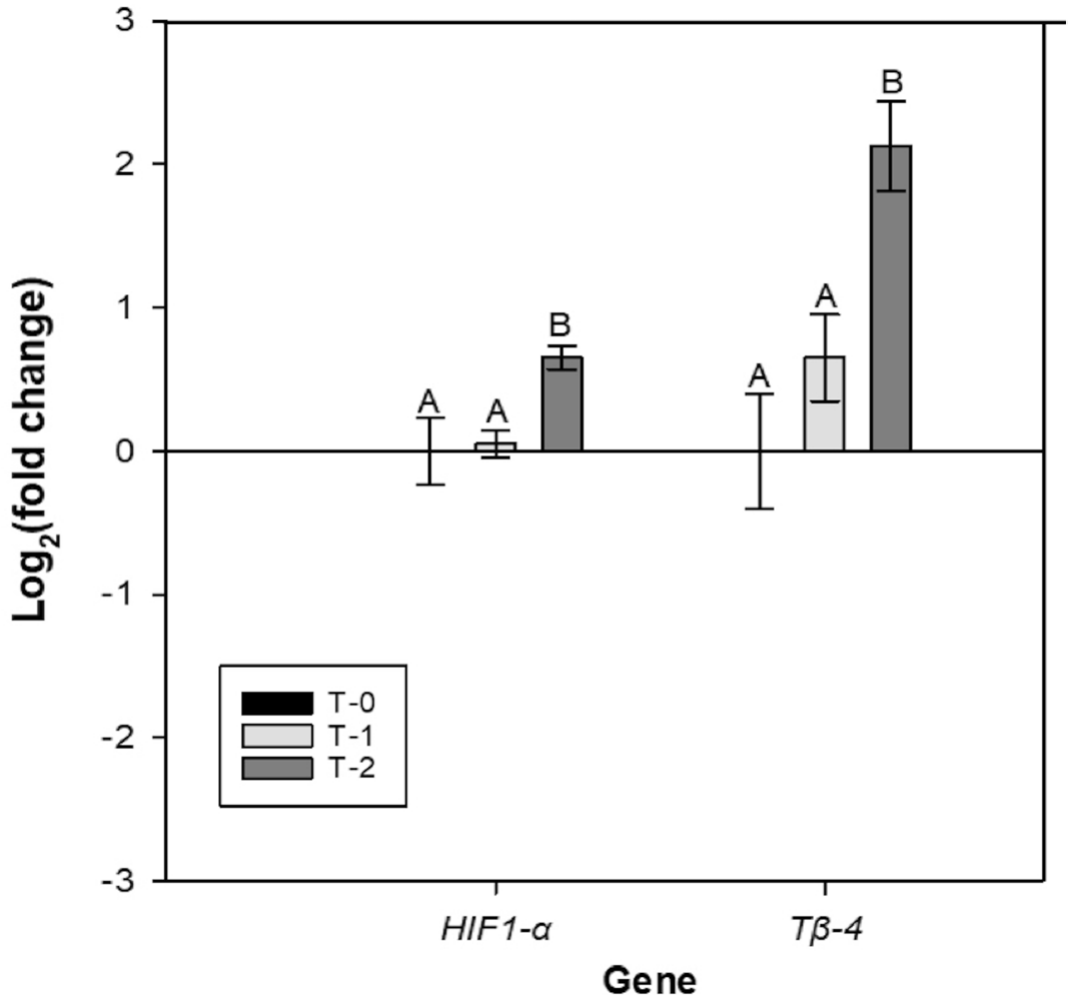
Relative mRNA expression of *HIF1-α* and *Tβ-4* for oysters sampled prior to deployment (T-0), on day 14 (T-1), and on day 21 (T-2) in the field deployment. A natural hypoxic event occurred on day 12 and lasted for the remainder of the deployment (Fig. 2). Bars represent means ± standard error (n = 5 oysters per time point). Letters denote times that were significantly different from each other based on Tukey’s post hoc tests (*HIF1-α*: p = 0.022 and 0.032 for T-0 vs. T-2 and T-1 vs. T-2, respectively; *Tβ-4*: p = 0.004 and 0.012 for T-0 vs. T-2 and T-1 vs. T-2, respectively).

Oyster gill *HIF1-α* mRNA expression showed a significant response to the natural hypoxic event (1-way ANOVA, F_2_ = 4.219, p = 0.044), with approximately 0.6-fold up-regulation on day 21 (after 9 days of hypoxia; T-2) compared to T-0 and T-1 oysters (Fig. 3). *Tβ-4* mRNA expression did not vary between oysters sampled at T-0 and T-1 (day 2 of hypoxia), but showed a significant twofold up-regulation in oysters following 9 days of exposure to a natural hypoxic event (1-way ANOVA, F_2_ = 9.08, p = 0.005; Fig. 3).

#### Total hemocyte counts

No significant differences in total circulating hemocyte counts were found among oysters collected over the course of the field deployment (mean ± 1SE: 3.86E^5^ ± 9.39E^5^, 4.24E^5^ ± 1.07E^5^, 5.0E^5^ ± 1.55E^5^ cells/mL for T-0, T-1, and T-2, respectively; 1-way ANOVA, F_2_ = 0.23, p = 0.798).

### Laboratory experiment 1: hypoxia-recovery

#### mRNA expression

**Figure 4:**
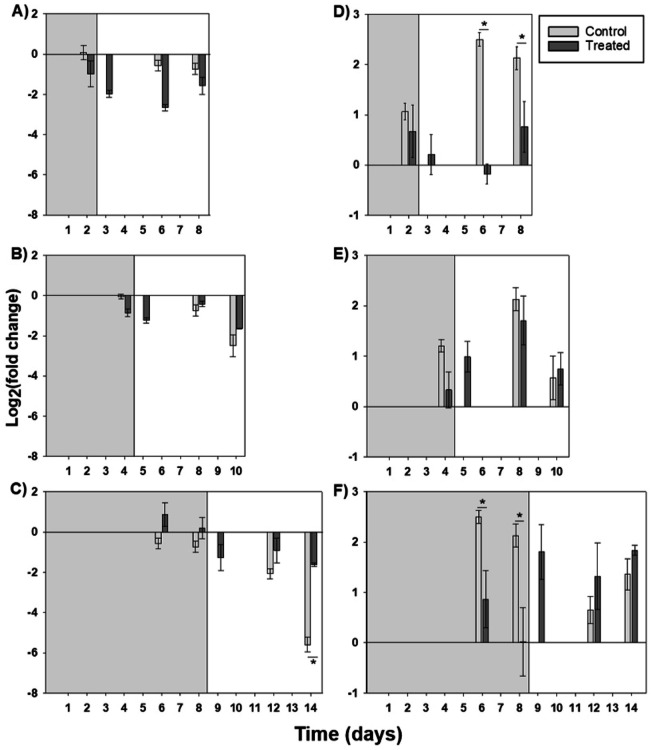
Oyster gill mRNA expression following different durations of hypoxia exposure (DO ≤ 2 mg/L), followed by a 6-day recovery period (DO ≥ 8 mg/L). *HIF-1α* mRNA expression during (**A**) 2, (**B**) 4, and (**C**) 8-day hypoxic exposures, and *Tβ-4* mRNA expression during (**D**) 2, (**E**) 4, and (**F**) 8-day hypoxic exposures. Bars represent means ± standard errors (n = 3 tanks per treatment). Grey background represents hypoxia period, white background represents recovery period. Asterisks denote significant differences between control and treatment determined via two-way ANOVA followed by a Tukey’s post hoc test (p < 0.05, Supplementary Resource 2).

Effects on oyster gill *HIF1-α* expression were measured after different durations of hypoxia exposure, followed by 6 days of recovery in the laboratory to assess onset and recovery of changes in gene expression. In the control treatment, *HIF1-α* and *Tβ-4* expression did vary significantly over time, and thus all data were normalized to the T-0 values. During the 2-day exposure, there was a significant main effect of time (p = 0.019) and treatment (p < 0.001) on *HIF1-α* expression (Fig. 4A; Table 3). In the 4-day exposure, there was a significant interaction between time and treatment (p = 0.022) for *HIF1-α* mRNA expression (Fig. 4B). Likewise, during the longest hypoxic exposure (8 days), there was also a significant time-treatment interaction (p = 0.01) in *HIF1-α* expression (Fig. 4C), with expression significantly more down-regulated in control oysters compared to treated on day 14 (i.e., after 8 days of hypoxia and 6 additional days of recovery) (p < 0.001; Supplementary Resource 2). Post hoc tests revealed a significant up-regulation of *HIF1-α* in treated oysters on day 6 of exposure compared to those on day 14, after 6 days of return to normoxia (p = 0.013). Acute changes (recovery) were not observed in *HIF1-α* expression in oysters following 24 h in normoxic conditions after hypoxia exposures of any of the three treatment durations (unpaired t tests, p > 0.05).

**Table 3:** Results from two-way ANOVAs on data from the laboratory experiments.

Experiment 1 – Hypoxia-Recovery										
Treatment	Response/Factor	df	F	p	Figure	Response/Factor	df	F	p	Figure
2-day hypoxic exposure	***HIF1-α expression***				4a	**Hemocyte counts**				5a
	Treatment	1	43.23	< 0.001*		Treatment	1	1	0.251	
	Time	3	4.208	0.019*		Time	2	3	0.095	
	Treatment × time	3	1.959	0.154		Treatment x time	2	1	0.383	
	***Tβ-4 expression***				4d					
	Treatment	1	41.87	< 0.001*						
	Time	3	2.811	0.067						
	Treatment × time	3	5.32	0.008*						
4-day hypoxic exposure	***HIF1-α expression***				4b	**Hemocyte counts**				5b
	Treatment	1	0.445	0.513		Treatment	1	6	0.035*	
	Time	3	11.02	< 0.001*		Time	2	0	0.925	
	Treatment × time	3	4.027	0.022*		Treatment × time	2	0	0.805	
	***Tβ-4 expression***				4e					
	Treatment	1	4.856	0.04*						
	Time	3	6.478	0.003*						
	Treatment × time	3	1.198	0.337						
8-day hypoxic exposure	***HIF1-α expression***				4c	**Hemocyte counts**				5c
	Treatment	1	26.19	< 0.001*		Treatment	1	11	0.007*	
	Time	4	17.34	< 0.001*		Time	3	4	0.03*	
	Treatment × time	4	4.236	0.01*		Treatment × time	3	1	0.311	
	***Tβ-4 expression***				4f					
	Treatment	1	2.137	0.157						
	Time	4	0.957	0.45						
	Treatment × time	4	4.025	0.013*						

Asterisks denote statistically significant main effect or interaction (p < 0.05).

After 2 days of hypoxic exposure, *Tβ-4* expression (Fig. 4D) showed a significant time by treatment interaction (p < 0.022; Table 3), with down-regulation compared to controls during each sampling point in the recovery phase (days 6 and 8; Supplementary Resource 2). After the 4-day exposure, *Tβ-4* mRNA expression (Fig. 4E) exhibited main effects of both treatment (p = 0.040) and time (p = 0.003). In the longest hypoxic exposure (8 days), *Tβ-4* gill expression showed a significant time by treatment interaction (p = 0.013) and was significantly down-regulated compared to controls on days 6 and 8 (Supplementary Resource 2). Within 24 h of return to normoxia, mRNA expression was similar to control levels (Fig. 4F).

#### Total hemocyte counts

**Figure 5:**
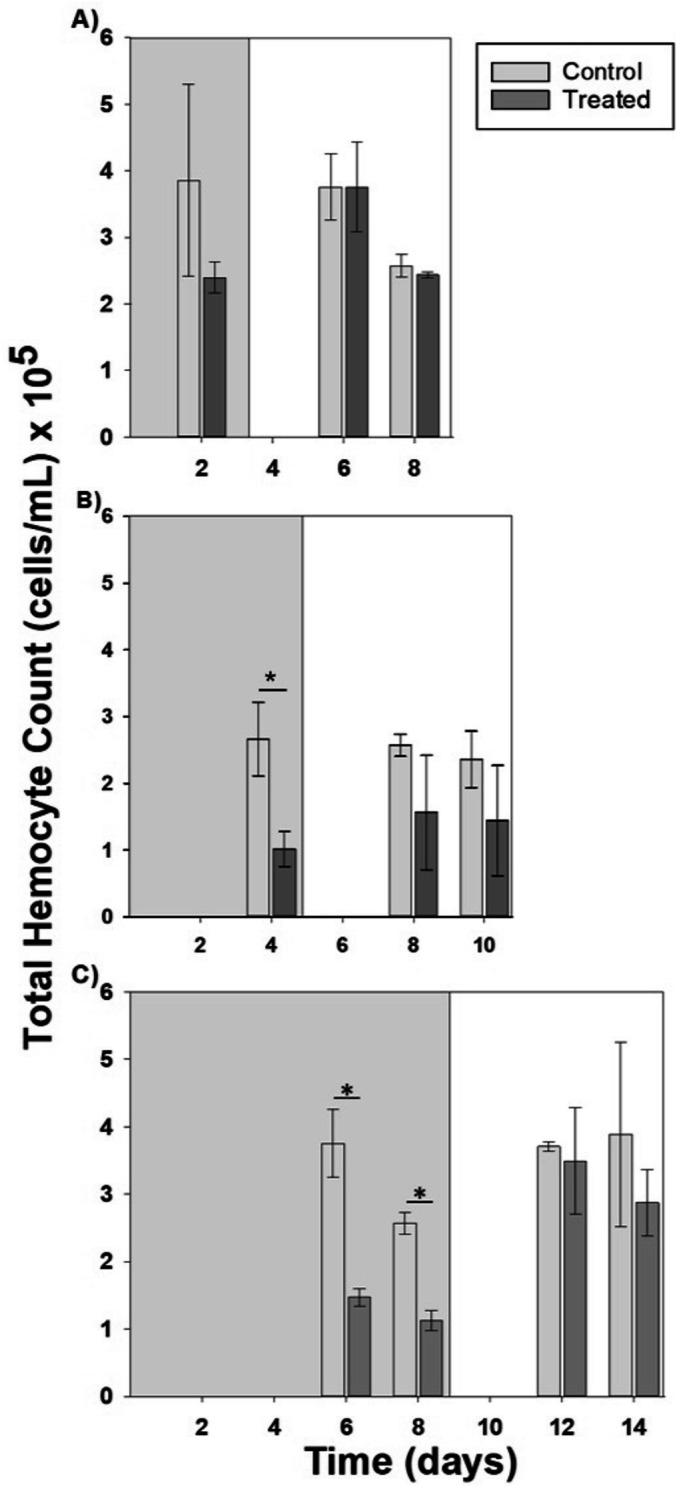
Total circulating hemocyte counts from hemolymph collected from oysters exposed to (**A**) 2, (**B**), 4, and (**C**) 8 days of hypoxia (DO ≤ 2 mg/L), followed by a 6-day recovery period (DO ≥ 8 mg/L). Bars represent means ± standard errors (n = 2–3 tanks per treatment). Grey background represents hypoxia period, white background represents recovery period. Asterisk denotes significant differences between treated and control oysters on that day, as determined using *t*-tests (p < 0.05).

In the 2-day hypoxic exposure, total circulating hemocyte counts did not differ across days or treatments (Fig. 5A; Table 3). Total hemocyte counts were significantly lower in exposed compared to control oysters after 4 days of hypoxic exposure (Fig. 5B, p = 0.035), but there was no significant time or interaction effect (Table 3). In contrast, during the 8-day exposure (Fig. 5C), hemocyte counts showed significant main effects of both treatment (p = 0.007) and time (p = 0.03), but no significant interaction (Table 3). In the longest hypoxic exposure, there was a significant reduction in total hemocyte counts in treated oysters compared to controls on days 6 and 8 of exposure to hypoxia (unpaired *t*-tests, p = 0.037 and 0.009, respectively).

### *Laboratory experiment 2: hypoxia* + *TBT*

#### Tissue accumulation of tin compounds

Oyster tissue collected at T-0 (n = 3) and from three oysters in each experimental treatment (n = 12) on day 8 of Experiment 2 was analyzed by B&B Laboratories for determination of mono-, di-, tri-, and tetra-butyltin concentrations (Table 2). All samples measured below the detection limit (< 9.95 ng Sn g^−1^ dry wt) for tetrabutyltins. Figures showing BT results can be found in Supplementary Resource 1.

#### mRNA expression

**Figure 6:**
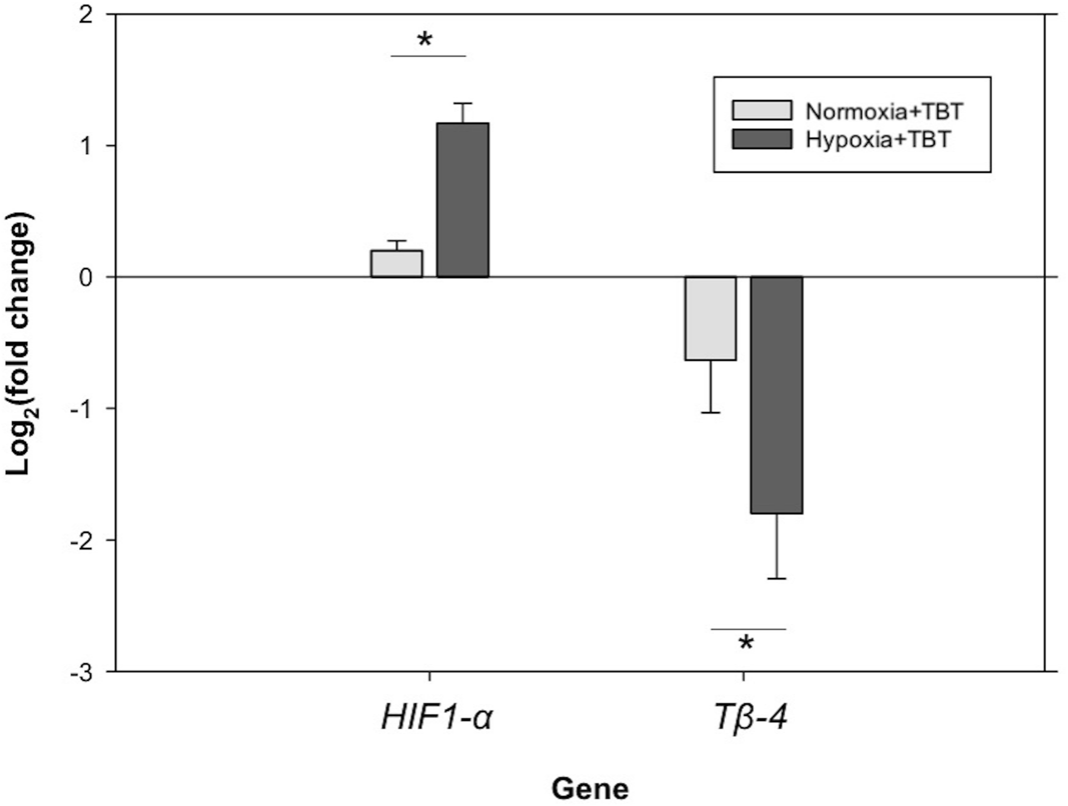
Relative mRNA expression of *HIF1-α* and *Tβ-4* for oysters exposed to 8 days of either normoxia + TBT or hypoxia + TBT treatments. Bars represent means ± standard error (n = 8 tanks per treatment). Asterisk denotes treatments that were significantly different from each other, as determined using unpaired *t*-tests (p < 0.05).

After 8 days of exposure, *HIF1-α* expression was significantly up-regulated (> twofold) in oysters in the combined hypoxia + TBT treatment compared to normoxia + TBT-treated oysters (two-tailed *t*-test, p < 0.001; Fig. 6). In contrast, there was a > twofold down-regulation of *Tβ-4* expression when exposed to hypoxia + TBT as compared to the normoxia + TBT treatment (Fig. 6, two-tailed *t*-test, p = 0.038).

#### Total hemocyte counts

**Figure 7:**
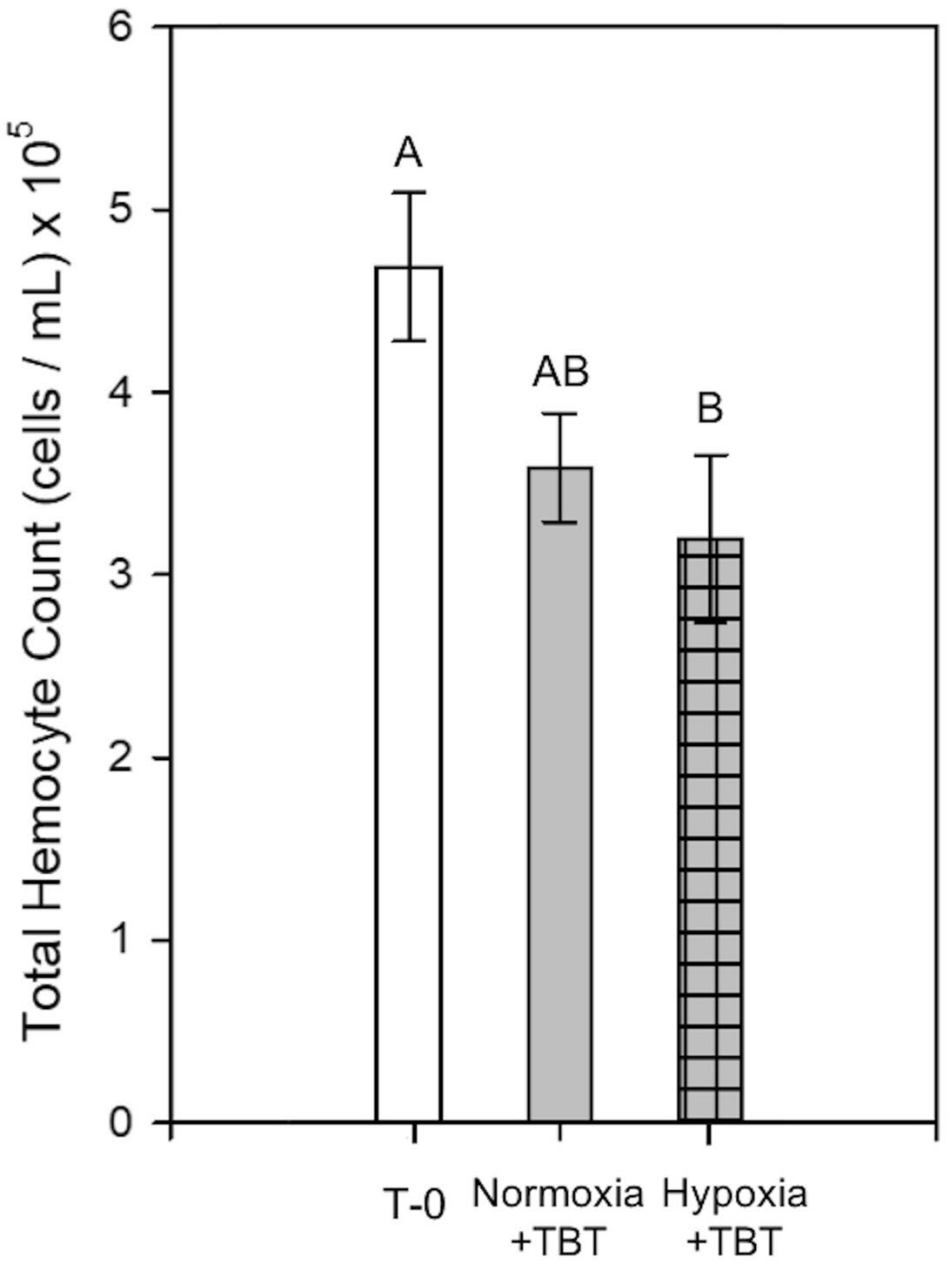
Total circulating hemocyte counts from hemolymph collected from oysters at T-0 and after 8 days of exposure to normoxia + TBT and hypoxia + TBT (Experiment 2). Bars represent means + standard errors (n = 2–6 tanks per treatment). Letters denote significant differences between treatments, as determined by one-way ANOVA (p < 0.05) and Tukey’s post hoc tests.

No statistically significant differences in hemocyte counts were observed for oysters in the normoxia + TBT treatment compared to the T-0 and hypoxia + TBT treatments after 8 days of exposure. Total circulating hemocytes decreased significantly in the combined hypoxia + TBT treatment when compared to T-0 oysters (Fig. 7, one-way ANOVA, p = 0.045).

#### Mortality

There were no mortalities in either the field deployment or Experiment 1. In Experiment 2, oysters in the hypoxia + TBT treatment had the highest mortality (2.5%) by day 8 of exposure. One oyster (0.6%) was lost in the normoxia + TBT treatment by day 8.

#### Prevalence of P. marinus cells

No *P. marinus* cells were found in any of the oysters sampled, indicating that oysters during experiments were not infected with Dermo disease.

## Discussion

Hypoxic events have become a common occurrence in estuarine environments and continue to increase in frequency and spatial scale^15,16^. Hypoxia can have detrimental effects on aquatic organisms and their populations, including suppressed immune function, reduced growth and reproduction, vulnerability to predation, and loss of biomass and biodiversity^17,37^. In the present study, in situ water quality sensors captured a hypoxic event that lasted throughout the final nine days of the field deployment near Marsh Point, MS (Fig. 2), although the full duration of this particular event is unknown. The random capture of this event during our brief field deployment points to a likely frequent occurrence of small-scale hypoxic events in the Mississippi Sound, an estuarine environment home to many fisheries species that are vital to the success of the coastal economy.

Due to their proximity to land and ever-increasing human populations, estuaries are subject not only to hypoxia, but to multiple stressors simultaneously. Environmental contaminants introduced into the environment through agricultural, storm-water, and sewage runoff can negatively affect and bioaccumulate in marine organisms^11^. Tributyltin, a biocide once ubiquitously used as an antifouling paint on marine vessels, causes calcification anomalies, and decreases survival in marine invertebrates^23^. Although largely banned by the early 2000s, TBT can reside in anaerobic sediment for years, with the potential for slow-release into the water column^38^. Hurricanes and coastal infrastructure development, such as port expansions, can facilitate resuspension of TBT-contaminated sediments. Due to the increasing occurrence of hypoxia alongside contamination, this study investigated the time-course of impacts of these stressors on molecular and physiological indicators of oyster health. We hypothesized that oysters subjected to longer durations of hypoxia would show signs of immunosuppression indicated by down-regulation of *Tβ-4* mRNA expression and decreased total hemocyte counts. Additionally, we expected that exposure of oysters to combined stressors, hypoxia and TBT, would elicit elevated stress responses compared to exposure to TBT alone. Lastly, we hypothesized that oysters exposed to hypoxia in the natural environment would exhibit similar stress responses to those found in laboratory experiments.

The gills are where gas exchange occurs first for the oyster, therefore, gill tissue was used based on previous studies where it was effective in showing expression of oxygenation-related stress biomarkers^31,32^. Increased *HIF1-α* mRNA expression is an indicator of hypoxic exposure. For example, in *C. virginica HIF1-α* was up-regulated after 6 days continuous hypoxia exposure^31^. In the present study, there was notable up-regulation in *HIF1-α* mRNA expression after 9 days in oysters that experienced a natural hypoxic event in the field compared to T-0 (1.6 fold change, Fig. 3). In Experiment 1, we expected to see an up-regulation in expression during exposure to hypoxia, followed by a decline during the recovery phase; the absence of this up-regulation could be explained by the large variance we observed in the controls. In Experiment 2, there was considerable up-regulation of *HIF1-α* in oysters exposed to combined hypoxia and TBT after 8 days of exposure when compared to normoxia + TBT (Fig. 6). We were unable to find evidence in literature of *HIF1-α* induction following exposure to TBT. Based on observation of *HIF1-α* expression, it appears that oysters exposed to hypoxia + TBT (Experiment 2) experience at least as much stress as those exposed to hypoxia alone (Experiment 1).

In all experiments, total circulating hemocytes, along with *Tβ-4* mRNA expression, were measured to assess potential for immunosuppression. Oysters possess a nonspecific immune response with phagocytic hemocytes acting as their main line of defense against pathogens^39^. The production of hemocytes is regulated by the Tβ-4 protein^32^, which is a recognized biomarker for immune-related stress^40^. In addition, *Tβ-4* demonstrates antimicrobial properties, as evidenced by its ability to suppress the growth of several strains of bacteria^41^. If there was stressor-mediated down-regulation in the mRNA expression of *Tβ-4*, it would be expected to cause a subsequent decrease in the number of total circulating hemocytes, resulting in immunosuppression. In previous studies where mussels were exposed to hypoxia, by 48 h of exposure, total hemocyte counts were substantially decreased^22^. Similarly, in our hypoxia-recovery experiment, total hemocyte numbers were reduced two-fold after 4 and 6 days of exposure and by half after 8 days, when compared to controls. Oysters appear to be resilient to hypoxia in their ability to produce hemocytes, because by 4 days of recovery, hemocyte numbers in treated oysters were not different than those of controls.

In Experiment 2, substantial down-regulation of *TB-4* was observed in oysters exposed to the combined hypoxia + TBT treatment after 8 days when compared to normoxia + TBT, supporting our hypothesis of elevated stress response under exposure to multiple stressors (Fig. 6). Additionally, total hemocyte counts were considerably lower in the hypoxia + TBT treatment when compared to T-0, while hemocyte counts in oysters exposed to normoxia + TBT showed no significant change (Fig. 7). Dermo, a disease caused by the pathogen *Perkinsus marinus*, is commonly associated with oyster mortalities and is likely to increase in distribution under warming ocean conditions^42^. TBT exposure negatively affects hemolymph quality in horseshoe crabs^43^, and hypoxia and TBT in combination increased the lethality of Dermo disease to oysters^28^. Although not the focus of this study, subsets of oysters from holding tanks and Experiment 2 were analyzed for Dermo disease to determine whether Dermo was a possible confounding factor in our experiments. No *P. marinus* cells were found in any of the oysters sampled. If Dermo had been present in our oysters, it is likely that the immune response and mortality would have been more exaggerated in treated oysters. Our results showing down-regulation of *Tβ-4* suggest that longer durations of hypoxia ( 6 days) and combined stressors may diminish the ability of oysters to fight pathogenic infection.

It should be noted that analysis of TBT accumulation in the tissue of laboratory-treated oysters showed rather high concentrations of dibutyltin (71–79% of total Sn) when compared to those of mono- and tri-butyltin (Table 2). In seawater, TBT has a short half-life of approximately six days, and typically undergoes photolytic or microbial degradation into di- and mono-butyltins^44,45^. Therefore, it is likely that TBT had already begun to degrade into its byproducts (mono- and di-butyltin) within the treatment tanks by 8 days of exposure, resulting in the bioaccumulation of higher percentages of mono- and di-butyltins relative to TBT concentrations. While dibutyltin exhibits immunologic toxicity in mammals, it is significantly less toxic to aquatic organisms than TBT^46^.

In the natural environment, hypoxic events can last for weeks or months and contaminated sediments can slowly release legacy contaminants, such as TBT, over long periods of time. The relatively short duration of our experiments and the use of adult oysters may have reduced the likelihood of observing dramatic effects of the stressors used in the present study. For example, juvenile and larval life stages are more sensitive to stressors than are adult oysters^14,21,25^. Whereas adult oysters can withstand anoxia for up to 28 days^47^, exposure to anoxia and hypoxia has detrimental effects on larval settlement^19^ and juvenile oysters exposed to severe diel cycling of hypoxia and pH experienced reduced growth^48^, which could negatively impact ecological functions, such as water filtration and resistance to predation. Spawning and larval recruitment are crucial to the maintenance and growth of oyster reefs, and oysters from metal-contaminated sites exhibit lower fecundity and reduced larval growth^49^. TBT is particularly toxic to early life history stages of oysters^25^, and has the potential to affect the success of nearshore oyster populations. We found that stressors in combination induced greater changes in mRNA expression when compared to the exposure of stressors alone. Indicators of immune suppression were adversely impacted by hypoxia exposure, both alone and in combination with TBT. Future studies of oyster reef health should take into account critical life stages, pollutants of growing concern, and other water quality parameters in relation to a changing climate.

## Supplementary information

Supplementary information.
