# Supplementary material for: Combined and independent effects of hypoxia and tributyltin on mRNA expression and physiology of the Eastern oyster (Crassostrea virginica)
Source: Sci Rep. 2020 Jun 30;10:10605. doi: 10.1038/s41598-020-67650-x (PMC7327041; doi:10.1038/s41598-020-67650-x)
Supplement: Supplementary file 1 — Supplementary information. [file 41598_2020_67650_MOESM1_ESM.docx]

Combined and independent effects of hypoxia and tributyltin on mRNA expression and physiology of the Eastern oyster (*Crassostrea virginica*)

Ann Fairly Barnett^a^†, James H. Gledhill^a^†, Robert J. Griffitt^b^, Marc Slattery^a^, Deborah J. Gochfeld^a,c^, Kristine L. Willett^a^*

†Joint co-authors contributed equally to manuscript

^a^ Division of Environmental Toxicology, Department of BioMolecular Sciences, University of Mississippi, P.O. Box 1848, University, MS 38677, USA

^b^ University of Southern Mississippi, School of Ocean Science and Engineering, Gulf Coast Research Laboratory, 703 East Beach Road, Ocean Springs, MS, 39564, USA

^c^ National Center for Natural Products Research, University of Mississippi, P.O. Box 1848, University, MS 38677, USA

*Corresponding author at: Division of Environmental Toxicology, Department of BioMolecular Sciences, University of Mississippi, P.O. Box 1848, University, MS 38677, USA kwillett@olemiss.edu (K.L. Willett)

E-mail addresses: afbarnet@olemiss.edu (A.F. Barnett), qcg2@cdc.gov (J.H. Gledhill), joe.griffitt@usm.edu (R.J. Griffitt), slattery@olemiss.edu (M. Slattery), gochfeld@olemiss.edu (D.J. Gochfeld)

**Supplementary information:**

**Supplementary Resource 1a-b: (A)** Mean concentrations of mono-, di-, and tri-butyltin in samples collected at T-0 (n=3), T-2 of field deployment (n=3), and in each treatment of Experiment 2 on day 8 (n=12). Bars represent means ± standard errors. All samples measured below the detection limit (<9.95 ng Sn g^-1^ dry wt) for tetrabutyltins. **(B)** Mean concentrations of tributyltin alone in samples described above. Bars represent means ± standard errors.

A)


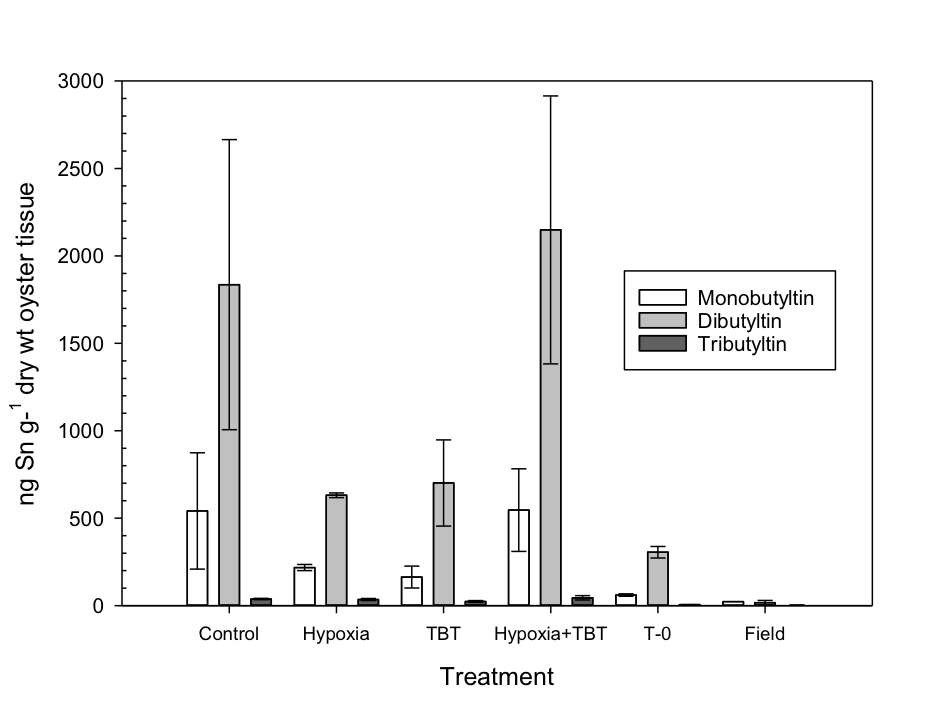


B)


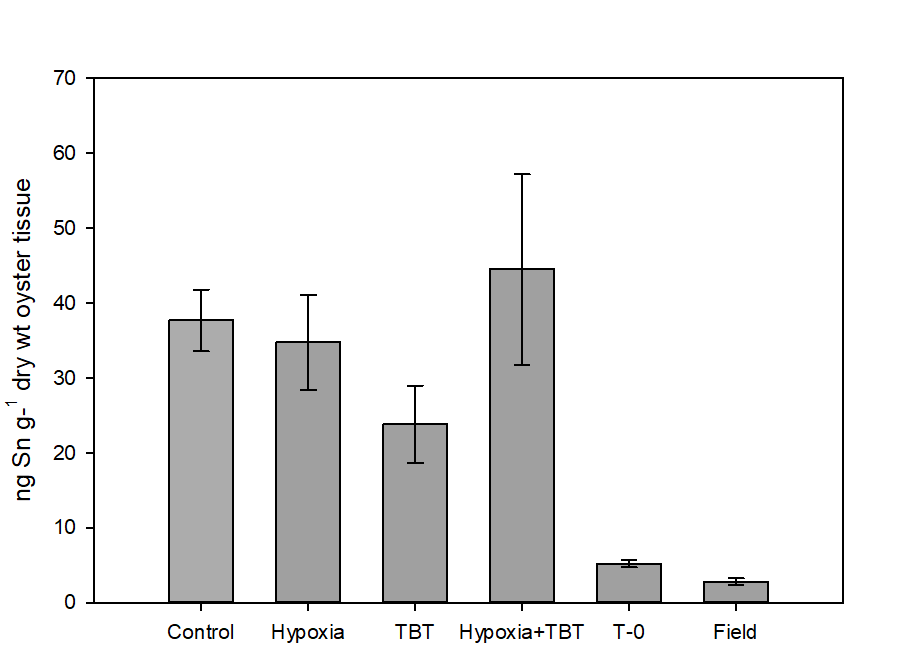


**Supplementary Resource 2:** Results of pair-wise post-hoc tests for significant main effect of time from 2-way ANOVAs on mRNA expression for Experiment 1: Hypoxia-recovery. Bold text indicates significant differences between a pair of days (p < 0.05) using Tukey’s post hoc test.

| Treatment/variable | Main effect | Comparison | P |
| --- | --- | --- | --- |
| 2-day exposure (*HIF1-α*) | Time | Day 2 vs. Day 6 | **0.012** |
|  |  | Day 2 vs. Day 8 | 0.128 |
|  |  | Day 6 vs. Day 8 | 0.525 |
| 4-day exposure (*Tβ-4*) | Time | Day 4 vs. Day 8 | **0.012** |
|  |  | Day 4 vs. Day 10 | 0.988 |
|  |  | Day 8 vs. Day 10 | **0.006** |
| 8-day exposure Hemocyte counts | Time | Day 6 vs. Day 8 | 0.466 |
|  |  | Day 6 vs. Day 12 | 0.303 |
|  |  | Day 6 vs. Day 14 | 0.463 |
|  |  | Day 8 vs. Day 12 | **0.039** |
|  |  | Day 8 vs. Day 14 | 0.061 |
|  |  | Day 12 vs. Day 14 | 0.979 |
